# The multidimensionality of social wellbeing: interactions from the individual to the collective level in large cities

**DOI:** 10.3389/fsoc.2023.1137797

**Published:** 2023-08-24

**Authors:** Oscar A. Martínez-Martínez, Araceli Ramírez-López, Eduardo Gamaliel Hernández Martínez, René Mac Kinney Romero

**Affiliations:** ^1^Department of Social and Political Sciences, Universidad Iberoamericana, México City, Mexico; ^2^Statistics Department, Colegio de Postgraduados, Texcoco, Mexico; ^3^Institute of Applied Research and Technology, Universidad Iberoamericana, México City, Mexico; ^4^Department of Electrical Engineering, Universidad Autónoma Metropolitana-Iztapalapa, México City, Mexico

**Keywords:** wellbeing, subjective wellbeing, community wellbeing, objective wellbeing, Mexico

## Abstract

This paper responds to two questions—What dimensions and indicators are relevant to the construction of social wellbeing? How are the levels of wellbeing distributed in the municipalities of Mexico City? To answer these questions, we use data from the Wellbeing Survey (*N* = 2,871) that is representative of Mexico City and its municipalities. We employed two methods, DM-R distances, and Mamdani's Fuzzy Inference Method. The results show that all the proposed dimensions and indicators contributed to the building of multidimensional social wellbeing; in the case of some indicators (social security, built environment, and public insecurity) they contributed less. This suggests government interventions should be designed in order to improve the gaps in those areas. The evidence also indicates that community wellbeing is a relevant dimension when measuring social wellbeing in large cities, in addition to identifying areas of intervention for the development of more efficient and inclusive public policies.

## Introduction

Wellbeing is a multidimensional, contextual concept, and its measurement has changed due to new needs or gaps in society (Grasso and Canova, 2008). There is no single definition (Galloway et al., 2006) but it is considered as an individual state resulting from people's life experiences (Kahn and Juster, 2002; Pollard and Lee, 2003). The dimensions that compose it and the way it is measured have evolved, reaching multidimensional measures (OECD, 2011). One of these dimensions is the objective or material wellbeing (OWB), made up of various components to satisfy essential human needs (OECD, 2011), such as income, health, education, employment, housing, and basic services, nutrition, and access to technology (Stiglitz et al., 2009; London et al., 2010) to point out a few.

Subjective wellbeing (SWB), another dimension, refers to the evaluations, both positive and negative, that people make of their life as a whole or in various domains (Diener, 2006; Stiglitz et al., 2009) and where the context plays a relevant role (Diener and Suh, 1997). SWB has generally been measured by satisfaction with life and happiness (Diener, 2000).

Regarding Community wellbeing (CWB), it is a dimension that has been gradually integrated into the literature due to its importance in the construction of wellbeing; some studies present it as the sum of variables that generate the SWB (Amérigo and Aragones, 1997; Liu et al., 2017); other scholars (EVALUA, 2017; Urbano et al., 2021), to which we subscribe, integrate CWB components and indicators as parts of multidimensional wellbeing. For instance, the CWB is composed of variables at the neighborhood or community level, such as social capital (Sarracino, 2013), public insecurity (Graham and Chaparro, 2011), the built environment, pollution, and social participation (Domínguez-Serrano and del Moral, 2018; Sabbadini and Maggino, 2018).

In previous studies, the OWB has been combined with the SWB to obtain general measures of multidimensional wellbeing (OECD, 2015, 2017), but only in a few studies CWB is also integrated (Martinez-Martinez et al., 2016; Urbano et al., 2021). This could be explained because when incorporating the three dimensions (OWB, SWB, and CWB), several subjective indicators at the micro and meso social level must be mixed with the objective indicators; this is an important challenge, but which is increasingly being carried out as part of synthetic measurements where they combine indicators of different levels (micro and meso and macro social) and that at the same time are objective or subjective (see, Whelan and Maître, 2005). Furthermore, previous studies (OECD, 2017, 2020) have used states, or sometimes municipalities, as a unit of analysis despite at the individual or household level there are heterogeneous conditions of wellbeing. In turn, the relevance of a category or indicator could be different from an analysis at the state or municipal level.

These considerations become even more significant for large cities or metropolises, such as Mexico City (CDMX), the most populated city in Mexico and one of the most crowded in the world (INEGI, 2015; WEF, 2019). The city presents heterogeneous characteristics, such as low poverty and high levels of human development (CONEVAL, 2021; PNUD, 2022). Regarding wellbeing, some OWB indicators show, for example, that 97.6% of people have potable water in their homes, 99.93% have sanitation, 100% of homes have electricity, 64.7% of people have food security, and 90.5% have access to public or private health service (ENIGH, 2020). At the same time, there are disparities between SWB indicators that show different realities. For example, the inhabitants rate on average 5.6 (on a scale of 0 to 10) n the satisfaction score with Safety in the City, 6.9 in score satisfaction with the City, and 7.5 in the satisfaction score with their neighborhood (ENBIARE, 2021). Similarly, within the CWB indicators, it has one of the highest percentages of perception of insecurity at the national level (83.2%) while having a high number of crime victims (ENVIPE, 2022), as well as one of the highest rates of air pollution (SINAICA, 2022). These indicators can be even more heterogeneous among the municipalities that integrate CDMX, hence the importance of investigating what happens within them and the way in which wellbeing is composed.

For this reason, it is central to investigate which dimensions and indicators are relevant in the construction of multidimensional social wellbeing in CDMX and their municipalities, in specific, when merging the OWB, SWB, and CWB. This is relevant to fill the gaps in the literature on wellbeing, by providing evidence on how to measure this construct through the three dimensions (in a synthetic indicator) and capture the complex living conditions of people in metropolises. Furthermore, a composite indicator could identify wellbeing by levels, showing how the population in CDMX is stratified. Hence, the paper answers two questions: What dimensions and indicators are relevant to build social wellbeing? How are the levels of wellbeing distributed in the municipalities of CDMX?

## Literature review

Wellbeing is a purpose that all people seek, regardless of socioeconomic status, religion, and race (Nanor et al., 2021). This construct is dynamic because it changes as new dimensions and indicators are incorporated (Noll, 2011). It also integrates at least three dimensions OWB, SWB, and CWB (Martinez-Martinez et al., 2016; Urbano et al., 2021) where objective and subjective indicators coexist (Marans and Stimson, 2011; Nanor et al., 2018), at micro and meso social levels (Whelan and Maître, 2005; Rodríguez and Martínez-Martínez, 2017) and where there is no clear border between the types of indicators. In addition, objective measures always present subjective judgments (Diener and Suh, 1997; Goerlich and Reig, 2021).

In the case of objective wellbeing, there is consensus on the dimensions that integrate it, such as education, basic household services, employment (Sabbadini and Maggino, 2018), pensions, working conditions (Escudero and Simón, 2012), leisure and free time (Domínguez-Serrano and del Moral, 2018), income, and health (Stucki and Bickenbach, 2019). In the case of health, it must be measured from different angles, such as mental health (Domínguez-Serrano and del Moral, 2018), comorbidities, and self-perception of health (Stucki and Bickenbach, 2019).

Similarly, there are other objective dimensions that have been integrated into the OWB measurement, such as access to technology—e.g., access to the internet, computers, and telephones (landlines, mobile, or both; García and Martín, 2010). Access to technology (e.g., Internet), can increase economic growth and improve the wellbeing of the poorest groups (Galperin and Viecens, 2017). In the same way, access to culture leads to positive results in wellbeing (Berigan and Irwin, 2011; Daykin et al., 2018) especially in contexts of insecurity and violence (Reyes-Martínez et al., 2021). In addition, cultural participation is associated with positive effects on physical and mental health (Cohen et al., 2006; Nenonen et al., 2014), it improves social cohesion by developing social networks, creating social capital and feelings of belonging and identity, as well as building individual identity and social relations (Bouder-Pailler and Urbain, 2015; Daykin et al., 2018).

Regarding subjective wellbeing, the literature shows different models that represent its structure and components (Lucas et al., 1996; Angner, 2010; Reyes-Martínez, 2021). These approaches have as overlapping points that SWB should be integrated of at least by positive and negative evaluations that people make of their lives and their affective reactions to their experiences (Andrews and Withey, 2012; OECD, 2013). Another coincidence is that its components must be analyzed in a disaggregated manner (Pollard and Lee, 2003; Stiglitz et al., 2009). In this sense, the measurement of SWB usually includes satisfaction with life and happiness (Kahneman and Krueger, 2006; Diener et al., 2009). The former refers to how a person evaluates his or her life in general, an evaluation that provides information about what he or she has lived through (Kim-Prieto et al., 2005). Happiness is the result of a balance between life experiences, both good and bad (Cieslik, 2015).

As for the community wellbeing, it is composed of the evaluation of different categories of the context that positively or negatively affect people and the community (Martinez-Martinez et al., 2018). Evaluations of CWB are performed directly by people, or indirectly by public organizations (e.g., the crime rate; Rodríguez and Martínez-Martínez, 2017). Having difficulties at the community level can affect family wellbeing and generate community stressors (Fraser et al., 2018). The dimensions that have been integrated as part of the CWB when measuring wellbeing vary in the literature, some of them are the built environment, public insecurity, social capital, and environmental pollution (Ramírez and Martínez-Martínez, 2017).

The importance of considering the built environment in CWB is supported by previous research (Jones et al., 2009; Sugiyama et al., 2019). Having a good built environment increases wellbeing because it facilitates access to food and services, which becomes more relevant as age increments (Liu et al., 2017). Other social benefits embrace a reduction in physical and mental stress (Hansmann et al., 2007; McPherson et al., 2011) and the promotion of social interaction (Coley et al., 1997).

Public insecurity, including victimization and perception of insecurity (Weaver and Clum, 1995), is another relevant measure because of its significant effects on community wellbeing (Martínez-Martínez and Martínez-Carreón, 2020). The perception of a high level of insecurity in the surroundings creates physiological adaptations, psychological reactions, and behavioral changes, reducing wellbeing at the individual and collective level (Doran and Burgess, 2011).

Social capital is another component of the CSW, composed of networks, norms, and trust. Social capital increases wellbeing (Berigan and Irwin, 2011) because it helps members of a society to act more efficiently to achieve common goals (Putnam, 2015). Social capital has positive results on the wellbeing of the home due to the ties that lead friends and neighbors to help each other, so the chances of being poor decrease. Similarly, being able to enjoy a pollution-free environment is necessary for community wellbeing (Domínguez-Serrano and del Moral, 2018) because reduction in air pollution, especially PM10 particles, is directly related to personal wellbeing (Mendoza et al., 2019). In other words, living in an environment with contamination reduces individual and collective wellbeing (Mendoza et al., 2019).

In sum, two main gaps are identified in the literature on social wellbeing. First, the integration of the CWB dimension when measuring multidimensional wellbeing, since most of the studies that have generated synthetic measures of wellbeing have only included OWB and SWB. Second, despite the boundaries of the objective and subjective indicators are not clear (Diener and Suh, 1997; Goerlich and Reig, 2021) it is possible to contribute to this clarification, in particular, when the variables are located at different levels (micro and meso social) and types (objective and subjective ones).

## Materials and methods

### Measures

The dimensions, subdimensions, indicators, and type indicators of multidimensional social wellbeing used in the analysis (see Table 1) were selected considering previous studies (OECD, 2015; EVALUA, 2017; Martinez-Martinez et al., 2018) that have used objective wellbeing with subjective wellbeing categories or with some indicators of community wellbeing. To see the more specific calculation of some indicators, consult the Supplementary material.

**Table 1:** Structure and measures of multidimensional social wellbeing.

**Dimension**	**Subdimensions**	**Indicator**	**Measure**	**Indicator type**
Objective wellbeing	Education	Lag in education	Percentage of household members with no lag in education	Objective
	Employment	Remunerated employment	Percentage of the economically active household population that worked the week before the survey	Objective
	Social Security	Medical services as a work benefit • Paid workers' compensation in the case of an accident, illness, or maternity leave • Access to a contributory or non-contributory retirement or pension system	Percentage of household members with access to social security	Objective
	Access to food	Food insecurity	Level of household food insecurity	Objective
	Quality of living spaces	Flooring material in the home • Roofing material in the home • Wall material in the home • Overcrowding	Quality level of household spaces	Objective
	Basic household services	Access to water in the home • Sewage in the home • Electricity in the home • Cooking fuel	Percentage of basic household services	Objective
	Income	Lag in income	Percentage of household members with no lag in income	Objective
	Health	Health services	Percentage of household members with access to health services	Objective
		Self-perception of health	Self-perception of health rating	Subjective
		Absence of comorbidities	Number of illnesses	Objective
		Depression	Depression scale	Objective
	Technology use	Computer	Access to a computer	Objective
		Internet	Access to Internet at home	Objective
		Landline	Access to a landline in the home	Objective
		Cell phone	Access to a mobile phone	Objective
	Access to culture and free time	Cultural events	Cultural events attended over the last six months	Objective
		Free time	Indicator that captures if one day a week free time is always and often had	Subjective
Subjective wellbeing	Satisfaction with life	Satisfaction with life level	Satisfaction with Life scale	Subjective
	Happiness	Happiness level	Happiness scale	Subjective
Community wellbeing	Social capital	Belonging to social networks	Number of groups belonged to	Subjective
		Trust in neighbors	Indicator that captures if there is trust in neighbors or not	Subjective
		Trust in institutions	Percentage of institutions trusted	Subjective
	Built environment	Constructed environment	Percentage of neighborhood infrastructure: streets with sidewalks, lighting, green spaces, crosswalks, and traffic lights	Subjective
		Clean streets	Indicator that captures if there is usually garbage in the neighborhood streets	Subjective
		Quality of transportation	Rating of public transportation	Subjective
	Pollution	Air quality	Percentage of days in a year when the concentration (24-hour mobile average) of particles less than 10 micrometers (PM10) is good or regular	Objective
	Public security	Safety in the neighborhood	Rating of neighborhood safety	Subjective
		Absence of homicides	Indicator that captures if in the family or group of friends someone has died as a victim of crime in CDMX	Subjective
		Absence of crime victims	Indicator that captures if a household member has been a victim of a crime in the neighborhood where he/she lives, or in any place in CDMX, over the past 12 months	Subjective

Source: Author's elaboration.

### The survey

Data employed in the analysis comes from the wellbeing Survey (Well-being Survey, 2017) carried out by the government of CDMX. It covers the whole city and each of its 16 municipalities. The sample design was probabilistic, multi-stage, stratified, and by conglomerate, using Mexico City's geopolitical division by municipality, which creates a natural stratification. Second, each municipality stratum was shaped by blocks with high, medium, and low levels, according to the CDMX Social Development Index (EVALUA, 2010).

In each municipality and stratum, conglomerates were formed of continuous or close blocks of houses, which made up the main units in the sample. The sample selection consisted of choosing conglomerates in each municipality and stratum, with proportional odds to the number of households in each. Alter that stage, the households were selected systematically, with equal odds. For each selected household, the head of the household or another adult was interviewed with informed consent. The data collection procedure was a paper-and-pencil interview, in which the interviewer used a paper questionnaire to read the questions and register the answers. A total of 2,871 households were surveyed with a rate response of 96%.

### Procedure and data analysis

According to Somarriba and Pena (2009), the most used methods to elaborate synthetic indicators are (a) the principal component analysis and (b) the data envelopment analysis. The method of principal component, although it is technologically straightforward and avoids duplicity of information, reports difficulties in the construction of indexes when variables are not very highly correlated. So, in this research, this method can exclude variables such as air quality, which is not highly correlated with other variables. Likewise, the data envelopment analysis uses linear programming in order to aggregate partial indicators; this method is flexible because it allows allocating different weights to partial indicators. However, this technique can assign zero or very low weight to specific indicators that, from a theoretical point of view, are very important.

On the other hand, the *DP*2 method generates synthetic indicators that have desirable mathematic properties like monotony and invariance to the chance of origin or scale of the variables. Besides these properties, the method allows the aggregation of variables expressed in different measures and avoids duplication of information (Somarriba and Pena, 2009).

Bearing that in mind, the method used in this study was the *DM* − *R* algorithm, derived from the *DP*2 distance technique (Somarriba and Pena, 2009). The contribution of the *DM* − *R* method lies in adding weights that consider the importance of the different indicators, unlike the *DP*2 method, which weighs all indicators in the same way. The subjective indicators were believed to have less weight because several studies (Martínez-Martínez et al., 2021) suggest that if there is no difference in weighting, the results tend to be overestimated. The indicators with the least weight were the subjective ones (see Table 1).

The *DM* − *R* method can be formulated as follows:

Let *X* = {*x*_*ij*_} the data matrix corresponding to *m* indicators (columns) in *n* territorial units (rows). In this case, households are considered as territorial units, such that *x*_*ij*_ is the value of the j-nth indicator in the *i*-th household.

The synthetic indicator for the *i*-th household is given as:


$$\begin{array}{l} DM-R_{i}=\overset{m}{\underset{j}{\sum }}\frac{d_{ij}}{\sigma_{j}}w_{j}\left(1-R_{j,j-1,j-2,..,1}^{2}\right);\;i=1,2,\;\ldots ,n. \end{array}$$

Where:

$d_{ij}=|x_{ij}-{x_{pj}}^{*}|$ is the distance between the *j*-nth indicator of the *i*-th household with respect to the reference point of the *j*-nth indicator, ${x_{pj}}^{*}$. In this case, ${x_{pj}}^{*}$ was considered as equal to the lowest value of the *j*-nth indicator in the data matrix.

σ_*j*_ is the standard deviation of the *j*-nth indicator.

$R_{j,j-1,j-2,..,1}^{2}$ is the determination factor in the linear regression of the *j*-th indicator (the independent variable) for indicators *j*−1, *j*−2, …, 1 (explanatory variables). It is assumed that $R_{1}^{2}=1$. The correction factor $\left(1-R_{j,j-1,j-2,..,1}^{2}\right)$ allows us to eliminate redundant information when we consider possible interdependence between indicators.

*w*_*j*_ is the weight associated with the *j*-nth indicator determined exogenously; it captures the importance of the *j*-nth indicator in building the index, taking on values from 1 to 0, where 0 is no importance, and 1 means very important. A weight equal to 0.5 was used in the calculation of the subjective indicators, and in other cases, a weight equal to 1 was used.

The results from the *DM* − *R* method for household *i* are equal to the sum of each indicator's contributions to wellbeing, where the contribution to the wellbeing of the *j*-nth indicator is the standardized distance between it and the reference value (the lowest value of that indicator in all households), corrected for redundant information in the indicator and weighted by its importance.

To calculate wellbeing levels we use Mamdani's fuzzy inference method (Lilly, 2011). This technique, unlike others employed in some studies (Zarzosa and Somarriba, 2013; OECD, 2017), performs an analysis of multiple subjective aspects that must be taken into account when mixing objective and subjective indicators. The advantages of fuzzy inference are its ability to understand and formalize social phenomena such as wellbeing through flexible models, and observing the subjectivity that arises from the perception of reality (García and Lazzari, 2001; Payán and Refugio Vallejo, 2015). We build a linguistic variable that defuzzifies the degree of membership of a household as a weighted average of the variables used in the measurement of wellbeing. Let *W* stand for the fuzzy set where any household *p*_*i*_ ∈ *W* has a degree of wellbeing in the *m* attributes of wellbeing *X* included in the survey.

Let,


$$\begin{array}{l} x_{ij}=\;\mu_{w}\left(X_{j}\left(p_{i}\right)\right),\;0\;\leq \;x_{ij}\;\leq 1 \end{array}$$

stands for the degree of membership to the fuzzy set *W*of the *i*-nth household (*i* = 1, …, *n*) with respect to the *i*-nth attribute (*j* = 1, …, *m*), such that, (i) *x*_*ij*_ = 1 if the *i*-nth household possesses the *j*-nth attribute; (ii) *x*_*ij*_ = 0 if the *i*-nth household does not possesses the *j*-nth attribute; and (iii) 0 < *x*_*ij*_ < 1 if the *i*-nth household possesses the *j*-nth attribute with a certain degree in the open interval (0, 1).

Let μ_*w*_(*p*_*i*_) stand for the wellbeing index of the *i*-nth household, i.e., the degree of membership of the *i*-nth household to the fuzzy set *W*.


$$\begin{array}{l} \mu_{W}\left(p_{i}\right)=\frac{\overset{m}{\underset{j=1}{\sum }}x_{ij}w_{j}}{\overset{m}{\underset{j=1}{\sum }}w_{j}} \end{array}$$

where *w*_*j*_ is the weight attached to the *j*-nth attribute, it is clear that,


$$0\;\leq \mu_{W}\left(p_{i}\right)\;\leq 1$$

which indicates the household *p*_*i*_ degree of membership to a multidimensional wellbeing index. A low value means a household has poor wellbeing while a high value means the household has good wellbeing. An inverted attribute has a degree of membership where greater values mean a lesser degree of membership and lesser values, a greater degree of membership. For these types of attributes we use the same weight adopted for normal values, but we use $x_{ij}=\left(1-{x^{{}^{\prime }}}_{ij}\right)$, where ${x^{{}^{\prime }}}_{ij}$ is the degree of membership for a normal attribute. Therefore, the degree of membership for a minimal value will be 1, and for a maximal value, 0, which is what we would expect.

The attributes that we consider subjective (see Table 1) are weighted down by using a weight that corresponds to double the maximal value present in the attribute. Therefore, the maximal degree of membership for a subjective attribute can only be 0.5. Given that the weights we adopt are fixed, it becomes,


$$\begin{array}{l} \mu_{W}\left(p_{i}\right)=\frac{\overset{m}{\underset{j=1}{\sum }}x_{ij}}{m} \end{array}$$

Note that μ_*W*_(*p*_*i*_) comprises all possible degrees of membership to the multidimensional social wellbeing index. We want to estimate the degree of membership as a linguistic variable, i.e., referring to μ_*W*_(*p*_*i*_) as low, medium, high, and very high. Dagum and Costa (2004) use α cuts (Zadeh, 1975) to obtain an estimate of a wellbeing ratio. We took a different approach by segmenting the values of membership obtained in quantiles, each containing 25% of *p*_*i*_ elements. Given the small distance between the degrees of membership, we tried different fuzzy sets to represent each of the linguistic variables. We obtained better results using a gaussian membership function for each of the quantiles. Finally, we defuzzified each μ_*W*_(*p*_*i*_) as an estimate of the multidimensional social wellbeing index.

## Results

Table 2 depicts the percentage of coverage in access to services and other social characteristics for the study context.

**Table 2:** Percentage of coverage in access to services and other social characteristics for each municipality.

**Municipality**	**Education**	**Social security**	**Access to food**	**Quality of living spaces**	**Basic household services**	**Income**	**Health services**	**Internet**	**Free time**	**Cell phone**	**Absence of homicides**	**Absence of crime victims**	**Air quality**
Álvaro Obregón	*93.86*	62.65	88.13	92.76	96.69	92.61	80.75	48.89	33.51	33.51	83.40	51.20	94.41
Azcapotzalco	94.75	68.15	92.05	92.58	96.58	90.07	84.88	67.72	35.25	35.25	86.14	43.31	88.72
Benito Juárez	98.22	74.62	95.39	95.37	100.00	97.44	81.88	81.62	37.45	37.45	83.67	41.63	94.45
Coyoacán	95.08	58.99	93.37	94.30	98.43	96.94	80.46	68.78	29.35	29.35	89.99	57.16	94.22
Cuajimalpa de Morelos	94.36	51.43	90.48	94.00	96.11	93.18	80.29	51.09	40.83	40.83	82.36	51.86	94.46
Cuauhtémoc	94.40	58.65	89.56	96.50	98.85	97.33	78.46	65.87	29.61	29.61	87.98	42.20	89.41
Gustavo A Madero	93.08	57.03	86.32	93.15	99.16	89.91	80.97	50.55	24.35	24.35	83.42	46.05	78.76
Iztacalco	95.70	58.71	90.98	91.17	98.48	92.20	78.52	64.58	37.26	37.26	92.14	34.31	93.80
Iztapalapa	92.33	56.62	85.11	91.26	95.65	89.29	76.68	57.11	31.94	31.94	86.67	43.46	86.90
La Magdalena Contreras	94.97	58.42	88.24	91.61	97.96	90.11	83.17	73.66	36.09	36.09	83.84	28.98	92.33
Miguel Hidalgo	96.88	64.46	95.95	92.34	98.72	97.39	82.12	49.93	27.93	27.93	77.51	39.15	92.28
Milpa Alta	91.63	40.56	85.71	91.83	87.94	86.05	77.50	29.73	11.25	11.25	93.52	44.16	94.46
Tláhuac	93.87	47.97	85.44	93.16	95.17	87.16	81.96	42.84	19.77	19.77	86.79	47.86	92.52
Tlalpan	92.78	48.65	86.53	89.70	91.28	89.85	77.44	50.33	24.04	24.04	78.74	45.19	93.08
Venustiano Carranza	97.36	61.04	89.92	94.14	98.12	92.48	82.13	48.87	30.30	30.30	81.41	21.91	85.82
Xochimilco	91.18	50.87	86.84	91.61	94.43	88.79	75.06	45.64	17.68	17.68	86.75	34.35	93.90

Source: Authors' elaboration, based on the WBS database.

Table 3 shows the indicators with which we build multidimensional social wellbeing. The weighted matrix is used as a measure that contemplates the standardized distance between the indicator and its lowest value in all households. After, it was corrected for redundant data in the indicator, multiplied by the correction factor, and finally weighted with 0.5 or 1.0 for each case.

**Table 3:** Contribution of indicators to multidimensional social wellbeing.

**Municipality**	**Quality and spaces in the home**	**Basic household services**	**Education**	**Employment**	**Depression**	**Self-perception of health**	**Air quality**	**Access to food**	**Health services**	**Absence of victims of crime**
Álvaro Obregón	9.88	9.24	4.96	4.79	3.60	3.65	3.36	2.60	3.07	2.32
Azcapotzalco	10.18	9.30	5.00	4.92	4.25	3.58	2.65	2.75	3.22	2.32
Benito Juárez	10.15	9.46	5.19	4.95	4.12	3.68	3.24	3.33	3.11	2.33
Coyoacán	10.21	9.34	5.02	4.93	4.05	3.83	3.31	2.88	3.06	2.34
Cuajimalpa de Morelos	9.99	9.28	4.98	4.89	3.99	3.63	3.44	2.78	3.05	2.22
Cuauhtémoc	10.26	9.41	4.99	4.98	4.09	3.66	2.72	2.82	2.98	2.28
Gustavo A Madero	10.18	9.41	4.92	4.77	4.10	3.61	1.04	3.01	3.08	2.31
Iztacalco	10.06	9.44	5.05	4.93	3.98	3.11	3.17	3.05	2.98	2.29
Iztapalapa	10.10	9.23	4.88	4.82	3.94	3.47	2.47	2.79	2.91	2.36
La Magdalena Contreras	10.23	9.36	5.02	4.87	4.16	3.26	3.43	3.19	3.16	2.21
Miguel Hidalgo	9.99	9.26	5.12	4.95	3.85	3.48	3.01	3.07	3.12	2.31
Milpa Alta	10.16	8.89	4.84	4.84	3.69	3.74	3.24	3.15	2.94	2.29
Tláhuac	10.04	9.23	4.96	4.77	4.06	3.96	2.94	2.97	3.11	2.44
Tlalpan	10.03	9.05	4.90	4.76	3.74	3.50	3.40	2.82	2.94	2.29
Venustiano Carranza	10.22	9.37	5.14	4.92	3.77	3.29	2.36	2.75	3.12	2.18
Xochimilco	10.11	9.20	4.82	4.78	4.10	3.30	3.18	2.83	2.85	2.35
**Municipality**	**Satisfaction with life**	**Absence of homicides**	**Happiness**	**Free time**	**Cell phone**	**Comorbidities**	**Constructed environment**	**Social security**	**Landline**	**Quality of transportation**	**Neighborhood safety**
Álvaro Obregón	2.26	1.99	1.95	1.60	1.36	1.37	1.12	1.24	1.09	0.90	0.88
Azcapotzalco	2.25	2.05	1.93	1.53	1.63	1.48	1.13	1.35	1.18	0.82	1.03
Benito Juárez	2.02	1.99	1.82	1.71	1.72	1.57	1.56	1.48	1.40	0.82	0.97
Coyoacán	2.30	2.14	1.98	1.56	1.57	1.50	1.38	1.17	1.29	0.91	1.10
Cuajimalpa de Morelos	2.33	1.96	1.99	1.61	1.45	1.36	1.36	1.02	1.17	1.01	1.21
Cuauhtémoc	2.17	2.09	1.88	1.60	1.51	1.41	1.45	1.16	1.11	1.09	0.87
Gustavo A Madero	2.22	1.99	1.89	1.52	1.55	1.46	1.25	1.13	1.12	1.07	0.82
Iztacalco	2.23	2.19	1.90	1.68	1.65	1.38	1.46	1.16	1.08	0.95	0.92
Iztapalapa	2.26	2.06	1.94	1.56	1.48	1.42	1.10	1.12	1.05	0.97	0.88
La Magdalena Contreras	2.27	2.00	1.90	1.63	1.82	1.59	1.09	1.16	1.44	0.99	1.02
Miguel Hidalgo	2.21	1.85	1.90	1.52	1.28	1.47	1.52	1.28	1.12	1.05	0.88
Milpa Alta	2.30	2.23	1.90	1.46	1.50	1.44	1.12	0.80	0.84	1.06	1.08
Tláhuac	2.28	2.07	1.99	1.52	1.32	1.46	1.41	0.95	0.96	1.02	0.88
Tlalpan	2.20	1.87	1.89	1.42	1.29	1.43	1.19	0.96	1.05	0.90	1.00
Venustiano Carranza	2.19	1.94	1.89	1.56	1.58	1.29	1.53	1.21	1.00	1.22	0.95
Xochimilco	2.11	2.06	1.75	1.37	1.49	1.45	0.92	1.01	1.04	0.90	0.98
Municipality	Internet	Trust in neighbors	Computer	Income	Cultural events	Belongs to social networks	Trust in institutions	Clean streets	Social wellbeing index
Álvaro Obregón	0.69	0.80	0.59	0.72	1.20	0.52	0.46	0.34	68.55
Azcapotzalco	0.96	0.85	0.75	0.77	0.70	0.44	0.45	0.31	69.78
Benito Juárez	1.16	0.83	1.00	1.38	1.17	1.16	0.52	0.47	74.31
Coyoacán	0.98	0.94	0.69	0.93	0.46	0.45	0.62	0.53	71.47
Cuajimalpa de Morelos	0.72	0.91	0.54	0.36	0.49	0.25	0.56	0.48	69.03
Cuauhtémoc	0.93	0.65	0.71	0.61	0.82	0.29	0.41	0.21	69.16
Gustavo A Madero	0.72	0.74	0.59	0.76	0.46	0.40	0.45	0.42	66.99
Iztacalco	0.92	0.68	0.83	0.69	0.34	0.73	0.48	0.45	69.78
Iztapalapa	0.81	0.77	0.75	0.53	0.96	0.42	0.51	0.35	67.91
La Magdalena Contreras	1.04	0.72	0.81	0.37	0.34	0.33	0.28	0.58	70.27
Miguel Hidalgo	0.71	0.80	0.67	0.86	0.47	0.40	0.41	0.42	68.98
Milpa Alta	0.42	0.66	0.71	0.35	0.48	0.27	0.28	0.47	67.15
Tláhuac	0.61	0.83	0.50	0.50	0.25	0.13	0.49	0.51	68.16
Tlalpan	0.71	0.80	0.65	0.59	0.51	0.60	0.39	0.48	67.36
Venustiano Carranza	0.69	0.72	0.71	0.59	0.52	0.84	0.42	0.45	68.42
Xochimilco	0.65	0.74	0.88	0.78	0.45	0.23	0.35	0.30	66.98

Source: Authors' elaboration, based on the WBS database.

Findings show that all indicators included in the measure contribute to building multidimensional social wellbeing but with different weights. The first five indicators with the highest average weight are from the objective wellbeing dimension. The first indicator was the quality of living spaces, with a contribution ranging from 10.23 in the La Magdalena Contreras municipality to 9.88 in Álvaro Obregón. The second indicator was basic household services, varying from 9.46 in Benito Juárez to 8.89 in Milpa Alta. The third one was education, from 5.19 (Benito Juárez), to 4.82 (Xochimilco). The next indicator was employment, with a weight of 4.98 (Cuauhtémoc) to 4.76 (Tlalpan). Next, depression varied from 4.25 (Azcapotzalco) to 3.60 (Álvaro Obregón). In sixth place was the self-perception of health indicator, ranging from 3.96 (Tláhuac) to 3.11 (Iztacalco). The last two dimensions are part of the health subdimension, which shows how important that division is to building wellbeing.

The rest of the indicators in the objective wellbeing dimension are distributed in different positions. For instance, access to food is in eighth place, followed by health services in ninth place. These two indicators show a very homogenous contribution in all municipalities. Comorbidities were in 16th place, and social security in 18th place. Many municipalities, such as Milpa Alta, Tláhuac, and Tlalpan showed social security contributions of <1. It means that there are fewer people covered in these areas. Income was in 25th—or last—place, except in Benito Juárez, which means that its contribution to wellbeing was minimal.

Free time is in 14th place, with the highest reading in Benito Juárez (1.71) and the lowest in Xochimilco (1.27), the region where people have the least amount of free time. Attending cultural events (26th place) was the last one in the objective measurement and is one that contributes least to wellbeing in all municipalities, which shows that households attend a limited number of cultural events.

Regarding technology use, the indicators showed different levels of contribution. The first was cell phones (15th place) and landlines (19th place). The municipalities with the highest indicators were Magdalena Contreras and Benito Juárez, meaning that they have more homes with landlines and more inhabitants with access to mobile phones. For Internet (22nd place), the municipalities with the highest contribution to wellbeing are Milpa Alta (0.42) and Xochimilco (0.65), and access to computers contributed the least in Tláhuac (0.50) and Cuajimalpa de Morelos (0.54), which can be explained by the low access to these indicators in these municipalities.

The subjective wellbeing indicators, such as satisfaction with life and happiness, are in 11th and 13th place in contribution to wellbeing, respectively. Satisfaction with life shows a level of contribution between 2.33 and 2.02, and the municipality where it contributes the most is Cuajimalpa de Morelos, and the least, Benito Juárez. Meanwhile, happiness varied from 1.99 to 1.75, and the municipality with the highest contribution again was Cuajimalpa, and the lowest, Xochimilco.

Results of the community wellbeing indicators had different contributions to the multidimensional wellbeing, but above all, they showed areas of intervention in public policies. The one that contributed the most to wellbeing was air quality, in 7th place, and Cuajimalpa de Morelos was where it contributed the most (3.44). Yet in municipalities such as Gustavo A. Madero (1.04) and Venustiano Carranza (2.36) it is possible to observe less contribution, which indicates that these areas have fewer days of the year with PM10 concentrations that fall in the good to regular range.

For the public security subdimension, what contributed the most was an absence of crime victims, in 10th place, with an average strength of 2.44–2.18. The second most important contribution was an absence of homicides, in 12th place, with a strength of 2.23–1.85. The contribution of both indicators in all municipalities was very homogenous. The indicator that contributed the least to wellbeing in this subcategory was security in the neighborhood, with an average strength in most municipalities of <1.0, which suggests a general perception of insufficient security among inhabitants in CDMX neighborhoods.

In the subdimension built environment, the constructed environment indicator (17th place) contributed the most on average to wellbeing, with a weight between 1.56 in Benito Juárez and 0.92 in Xochimilco. Quality of public transportation (20th place) contributed between 1.22 in Venustiano Carranza, and 0.82 in Azcapotzalco and Benito Juárez. Clean streets were the last indicator of all the dimensions, <1 in all municipalities and ranging from 0.58 in Magdalena Contreras to 0.21 in Cuauhtémoc, which shows that there is a homogenous perception of the lack of clean streets in the city. Results in this subdimension indicate that there is a better perception of street infrastructure, such as sidewalks, lighting, and green areas, than of the quality of public transportation or clean streets.

The indicators in the subcategory social capital tend to contribute little on average. Trust in neighbors is in 23rd place, ranging from 0.94 in Coyoacán to 0.65 in Cuauhtémoc. The indicator belonging to networks is in the 27th place, the highest in Benito Juárez (1.16), and the lowest in Tláhuac (0.13); these data show a gap between the number of social groups people belong to in these municipalities. Trust in institutions, in the 28th place, contributes on average <0.62, indicating the low percentage of trust in the different government and institutional bodies.

The above findings allow us to understand the results of the social wellbeing index found at the end of Table 3; which is a synthetic index that integrates in a single condensed measure, the three dimensions OWB, SWB, and CWB. The results show that the distance in the ranking between the different municipalities is very close, even between the first and last place. In this sense, the three places with the highest social wellbeing are Benito Juárez, Coyoacán, and La Magdalena Contreras; those that are in the final part of the index are Milpa Alta, Gustavo A Madero, and Xochimilco.

Table 4 provides information on how multidimensional social wellbeing (OWB, SWB, and CWB) is distributed in each municipality, in such a way that it can be observed what percentage of households are in each of the four levels (very high, high, medium and low). The municipalities where the majority of households are at the 'very high' social wellbeing level are Coyoacán (47.54%), Cuajimalpa de Morelos (47.06%), and Benito Juárez (46.00%). The municipalities with the highest percentage of 'high' social wellbeing households are Iztacalco (32.80%), Venustiano Carranza (30.89%), and Xochimilco (30.56%). In Benito Juárez and Coyoacán, more than 75% of the households are in the categories of very high and high wellbeing, this allows us to understand why in the multidimensional index of social wellbeing (see Table 3) they occupy the first places.

**Table 4:** Distribution of the levels of social wellbeing by municipality (%).

**Municipality**	**Very high**	**High**	**Medium**	**Low**
Álvaro Obregón	21.29	19.80	28.71	30.20
Azcapotzalco	27.48	27.48	29.01	16.03
Benito Juárez	46.00	29.33	16.00	8.67
Coyoacán	47.54	27.87	14.21	10.38
Cuajimalpa de Morelos	47.06	22.35	18.82	11.76
Cuauhtémoc	21.91	22.47	34.83	20.79
Gustavo A Madero	14.29	25.09	23.00	37.63
Iztacalco	35.20	32.80	18.40	13.60
Iztapalapa	16.25	23.00	26.50	34.25
La Magdalena Contreras	21.43	25.00	25.00	28.57
Miguel Hidalgo	33.60	24.80	28.80	12.80
Milpa Alta	9.30	20.93	11.63	58.14
Tláhuac	15.96	23.40	32.98	27.66
Tlalpan	25.60	19.05	21.43	33.93
Venustiano Carranza	26.02	30.89	28.46	14.63
Xochimilco	3.70	30.56	35.19	30.56

Source: Authors' elaboration, based on the WBS database.

Xochimilco has the highest percentage of households in the “medium” level of wellbeing (35.19%), followed by Cuauhtémoc (34.83%) and Tláhuac (32.98%). In Milpa Alta, almost 60% of households are in the “low” level. Other municipalities with high percentages of households in the “low” level were Gustavo A Madero (37.63%), Iztapalapa (34.25%), Tlalpan (33.93%), and Xochimilco (30.56). Almost all of these municipalities are located in the last places in the multidimensional index of social wellbeing.

## Discussion

Findings suggest that social wellbeing is a multidimensional construct composed of objective, subjective, and community wellbeing components, where subdimensions interact as part of a complex system. It means that social wellbeing should not be measured unidimensionally, but in a multidimensional way (Nanor et al., 2021) and that its dimensions are constantly changing, and new ones could be added according to the needs of society. The results of the measurements suggest that all dimensions, sub-dimensions, and indicators contribute to the proposed wellbeing construct but, unlike other studies where objective and subjective wellbeing have been combined (Khadka, 2019; Mehdi, 2019), the inclusion of community wellbeing provides solid evidence of the importance of including it when measuring social wellbeing in a multidimensional way. This has implications for the design of public policies, mostly at the neighborhood level.

The contributions of the dimensions and indicators to social wellbeing are different (see, e.g., Table 3). A high contribution to wellbeing means that the government's actions in terms of public policies are strong; contrary, low contributions suggest that it is necessary to design or reinforce public policies or social programs to improve these indicators and thus improve the population‘s wellbeing. In this sense, some subdimensions contributed the most to social wellbeing in all municipalities, such as quality of living spaces and basic household services. It is possible to explain this result by the fact that, when compared to other states, CDMX has high levels of services coverage, such as sewage, water, and electricity, and a low percentage of homes with low-quality roofs, floors, and walls (CONEVAL, 2021). Therefore, both are essential to building wellbeing in this context. The same can be observed in education and employment, two aspects are relevant to wellbeing, as some studies have shown (Domínguez-Serrano and del Moral, 2018; Sabbadini and Maggino, 2018).

In education, the gap in the lag at the national level has been reduced, particularly in CDMX, where we find the lowest percentage of people with no elementary schooling (CONEVAL, 2021). Moreover, employment is one of the indicators that most contribute to wellbeing, but at the same time is one of the most fragile in the face of economic crises, such as the one generated by the COVID-19 pandemic, which led to the loss of millions of formal and informal jobs and the uprising in unemployment (ENOE, 2021, 2022).

One relevant finding is that in the wellbeing ranking (see Table 3), the difference between Benito Juárez, the highest, and Xochimilco, the lowest, is barely a few units, even though other studies in other contexts (García and Martín, 2010; Martinez-Martinez et al., 2018) have shown that the inequality in wellbeing between the highest and lowest regions is very high, at times reaching three or four times the distance between the two. In CDMX these gaps might be explained because the contribution of almost all the indicators is quite homogenous, yet not all contribute strongly; some contribute in the same small amount in all municipalities, showing the importance of designing public policies that improve the conditions in the constructed environment, quality of transportation, neighborhood safety, trust in neighbors, and clean streets, since these are relevant in the construction of multidimensional social wellbeing as shown by various investigations (Liu et al., 2017; Nanor et al., 2021).

Meanwhile, some indicators suggest heterogeneous contributions and could highlight the differences in the way percentages of the levels of wellbeing are distributed within each municipality (see Table 4). They include two belonging-to-community wellbeing variables. The first is the subdimension of pollution, since, according to other research (Domínguez-Serrano and del Moral, 2018; Mendoza et al., 2019), having a pollution-free environment is necessary to achieve community wellbeing. Yet, the results show great heterogeneity between the municipalities in the air-quality index. Data even show that these differences are >3 times the distance between the territories with the highest and lowest air pollution (see Table 3).

Second, belonging to social networks supports that in certain contexts (for example Tláhuac, Xochimilco, Cuajimalpa de Morelos, and Milpa Alta) people do not tend to get involved in neighborhood, religious, cultural, or sports groups in the community. It could be due to the breakdown of the community's social fabric, much of this due to widespread mistrust, both with neighbors, unknown people, public institutions, and community groups -including religious and community ones- (Rodríguez-Brito, 2023); this derived from insecurity and criminality in certain areas of the city (Olvera Aldana and Martínez, 2019), and to people's free time, affected by the distance between work and the community, traffic, and complications in work life that are common in a city as large as CDMX (Rodríguez-Brito, 2018).

Income, access to culture, and Internet access presented large inequalities among municipalities, suggesting government intervention in these areas. Some of the main aspects that could explain why income contributed little to the multidimensional construction of social wellbeing (see Table 3), first, is that the percentage of the population with an income below the poverty line and the extreme poverty line has been high over time (CONEVAL, 2021). It implies high percentages of people who do not have enough income to cover the cost of food and non-food basic goods (CONEVAL, 2021). Second is weak economic growth and the low impact of social programs granting cash transfers (Martínez-Martínez et al., 2020). Hence the importance of improving social policy in terms of income, since Income is one of the essential aspects to achieve wellbeing (Domínguez-Serrano and del Moral, 2018) because it is how people pay for the goods and services they need at home.

Access to culture is paramount to building wellbeing, and it creates a better quality of life (Cohen et al., 2006) and social development (Vich, 2014) because it is related to physical and mental health (Grossi et al., 2011; Nenonen et al., 2014). Yet the CDMX study, like others, shows that access is unequal and linked to poverty or marginalization (Reyes-Martínez et al., 2021). We found that people in the municipalities farthest from downtown report attending fewer cultural events (for example Tláhuac, La Magdalena Contreras, and Iztacalco). This can be explained first by the distance and cost to get to the places where most cultural events are produced, and second by the lack of cultural offers in the municipality, as other studies have shown (Bouder-Pailler and Urbain, 2015; O'Brien and Oakley, 2015).

Internet access improves wellbeing, especially among the poorest (Galperin and Viecens, 2017) while increasing social capital by strengthening bonds with friends and family members. It is also important for work, making it, as well as other daily tasks, easier (Overå, 2006; Avilés et al., 2016). In CDMX, there is still inequality in household Internet access, even when access achieved through mobile phones becomes more common (ENDUTIH, 2021). The result for this indicator (see Table 3) suggests inequalities in access to Internet connectivity among households. This is a problem, especially because people need the Internet at home, as the COVID-19 pandemic revealed since they use it both for children's homework, online jobs, and even entertainment. Not having Internet in the home increases the digital divide (Leidig and Teeuw, 2015), while becoming a factor of social exclusion. Our findings also showed that the SWB indicators, satisfaction with life and happiness, have a relevant contribution to the construction of multidimensional social wellbeing. This is probably because of their combination with the indicators of the OWB and CWB dimensions.

In this sense, our results show that by incorporating the sub-dimensions and indicators of CWB, with those of OWB and SWB, more robust measurements are generated that integrate objective and subjective indicators at the micro, meso, and macro social level. Hence the relevance of integrating this dimension to measure social wellbeing in a multidimensional way, considering different areas of the life of human beings and their environment.

## Conclusions

Wellbeing is a construct composed of three large dimensions—OWB, SWB, and CWB. Each encompasses subdimensions and objective and subjective indicators that interact in the different dimensions. The contribution of the method to measure wellbeing by mixing these three dimensions, and particularly by including community wellbeing, is that it helps to identify areas of intervention for the development of more efficient and inclusive public policies. It is important to note that maintaining levels of wellbeing requires the ongoing implementation of different public-policy measures. Especially, in some municipalities where more than 50% of households are between medium to low wellbeing (see Table 4), such as Milpa Alta, Xochimilco, Tláhuac, and Iztapalapa, to name a few. In the same sense, there are others where most of the households have deficiencies in social wellbeing, both OWB, SWB, and CWB, for which several are at a low level of wellbeing, such as Milpa Alta where more than 50% of households are in that situation.

At the same time, in CDMX some indicators contribute greatly to wellbeing since they have improved over the years with government investment, in areas such as quality and space in the home, basic household services, health services, and education. However, others such as internet access, free time, access to cultural events, the built environment, social security, and public insecurity, have shown that more efficient public policies or the design of government interventions are needed to improve them, especially since all these categories and indicators were exacerbated as a result of the COVID-19 pandemic.

Findings lead to three public policy implications. First, the indicators that present higher contributions to wellbeing are fragile in the presence of economic and social problems, such as employment, access to food, depression, and self-perception of health, all of which can have an effect through an increment in public insecurity, reduction of social capital, less satisfaction with life, and less happiness. Ongoing investment is needed through social programs such as unemployment insurance, food assistance, and prevention programs regarding physical and mental health, as well as a significant investment in the health system to improve quality and coverage.

Second, indicators with lower contributions in all contexts, such as social security, the constructed environment, clean streets, and quality of transportation, require the design of public policies to improve wellbeing. Therefore, formal employment and benefits should be improved, and likewise, direct government investment is needed to improve neighborhoods. Third, indicators with low and unequal contributions require a major investment, through robust programs such as basic universal income, universal Internet access, and universal access to culture. Although these are actions that require significant investment by the government, this could be done in stages, especially in the first phase, covering people in poverty as well as the most vulnerable groups.

Finally, this study presents two limitations. First, even though the survey is representative of CDMX and its municipalities, it is a cross-sectional one. A significant challenge is to get information periodically to longitudinally compare wellbeing and find out the effects of social programs on the strengthening of wellbeing, or the interventions needed. Second, although the condensed measures allow adding different dimensions and indicators in a single index, face the challenge of trying to explain complex and multidimensional realities (Goerlich and Reig, 2021), which is not always achieved, in particular when the individual contribution of the indicators not is known; in our case, the techniques used made it possible to overcome this barrier, since both techniques helped to robustly condense all the indicators and subsequently, to know the individual contribution of each indicator to the multidimensional wellbeing.

## Data availability statement

The raw data supporting the conclusions of this article will be made available by the authors, without undue reservation.

## Ethics statement

The studies involving human participants were reviewed and approved by Universidad Iberoamericana. The patients/participants provided their written informed consent to participate in this study.

## Author contributions

OM-M, AR-L, and RM contributed to conception, design, and finalization of the paper. OM-M and AR-L contributed to drafts of the manuscript with special emphasis on social-science sections and DM-R Distances Method. EH and RM contributed the Mamdani's Fuzzy Inference Method. All authors contributed to manuscript revision, read, and approved the submitted version.
