# Supplementary material for: The multidimensionality of social wellbeing: interactions from the individual to the collective level in large cities
Source: Front Sociol. 2023 Aug 24;8:1137797. doi: 10.3389/fsoc.2023.1137797 (PMC10484406; doi:10.3389/fsoc.2023.1137797)
Supplement: Supplementary file 1 [file Data_Sheet_1.docx]

Supplementary Material

**The Multidimensionality of Social Well-Being: Interactions from the Individual to the Collective Level in large cities**

**Oscar A. Martínez-Martínez**^1*^, **Araceli Ramírez-López**^2^,**Eduardo Gamaliel Hernández Martínez**^3^**, René Mac Kinney Romero**^4^

^1^ Department of Social and Political Sciences, Universidad Iberoamericana, México City, México

^2^ Colegio de Postgraduados, Texcoco, México

^3^ Institute of Applied Research and Technology, Universidad Iberoamericana, México City, México

^4^ Universidad Autónoma Metropolitana-Iztapalapa, México City, México

*** Correspondence:**Oscar A. Martínez-Martínez
oscar.martinez@ibero.mx

**Indicators and Measures**

1. Education: Percentage of the population by age that has studied basic compulsory education^[[1]](#footnote-1)^ or is going to a formal education center (CONEVAL, 2014).
2. Social Security: Percentage of the population working and not economically active, and those 65 or older with access to social security^[[2]](#footnote-2)^ (CONEVAL, 2014).

Food insecurity: The level of household food insecurity was determined with the Latin American and Caribbean Food Insecurity Scale. Then, a dichotomous variable was built with a value equal to 1 when the household had food security or slight food insecurity, and equal to 0 when the household had moderate or severe food insecurity.

Quality level of household spaces: The indicators were calculated for lack of flooring material in the home, lack of roofing material in the home, or lack of wall material in the home and overcrowding, according to the CONEVAL (2014) methodology; the indicator quality of spaces in the home is equal to the percentage of these indicators where nothing is lacking.

Percentage of basic household services: The indicator lack of access to water in the home, lack of sewage in the home, lack of electricity in the home, and lack of cooking fuel (CONEVAL, 2014) were determined; the indicator for basic household services is equal to the percentage of these indicators were nothing is lacking.

Percentage of household members with no lag in income: The indicator was determined with the methodology from EVALUA (2017), where total current household income was calculated as ${ICT}_{h}$, in other words, current monetary income plus current non-monetary income; likewise, the number of household members, $N_{h}$ uses the equivalency scale mentioned in this document. The income indicator was determined as

$\left\{ \begin{aligned} 100\% si \frac{{ICT}_{h}}{LB}\geq N_{h} \\ \frac{{ICT}_{h}}{N_{h}\times LB}\% si \frac{{ICT}_{h}}{LB}< N_{h} \end{aligned} \right.$ , where $LB$ is the value of the welfare line, which is equal to the total value of the basic food and non-food essentials for the month of November 2017, or U.S.$132.65.

Health Services: The percentage of the population covered or with the right to receive medical care from a public or private institution (CONEVAL, 2014).

Self-perception of health: It is the answer to the question: In general, how would you rate your health? Very good, good, regular, bad, or very bad?

Absence of comorbidities: They are diseases that have already been diagnosed by a doctor or specialists, including diabetes, hypertension, obesity, high cholesterol, heart attack, cancer, liver disease, heart disease, kidney disease

Depression: The depression scale by the Center for Epidemiological Studies was used in its abbreviated version with 7 questions (Salinas- Rodriguez et al., 2013); the scale goes from 0 to 21 points total. A score of 9 or higher was used to determine depression. The indicator “absence of depression” used in this document has a value of 1 when the participant does not suffer from depression, and 0 if he or she does.

Cultural Events: It reports how many times the person attended dances, concerts, or music shows, plays, art exhibitions, visual art shows and cultural activities; or how many times the person visited a museum, library, archeological zone, historical monument, or cultural center.

Free Time: It is a dichotomous response by the participant to the question: On a normal work day, do you often have free time? A 1 is for “always” or “often” and a 0 is “not often” or “never.”

Satisfaction with Life level: It is the response to the question: How satisfied are you with your life?” on a scale of 1 to 10, where 1 means “not at all satisfied” and 10 is “very satisfied.”

Happiness level: It is the answer to the question: How happy are you? On a scale of 1 to 10, where 1 is “not happy at all” and 10 is “very happy.”

Belonging to Social Networks: It is the number of groups the person belongs to of these eight: religious, friendship (school, work, or both), professional association, athletic activities (cultural or artistic), neighborhood, political, unions, or others.

Trust in Neighbors: It is a dichotomous response to the question: Can I trust my neighbors? where 1 is yes and 0 is no.

Trust in Institutions: The percentage of the following institutions which the person trusts “a lot”, “somewhat”, “a little”, “nothing”: church, police, political party, army, federal government, Chamber of Deputies, press, government of CDMX, government of the delegation, banks, television, and the National Elections Institute.

Constructed Environment: First, the lack of sidewalks, lighting, green spaces, and crosswalks was considered; the indicator quality in the area is equal to the percentage of all these indicators where there is nothing lacking.

Quality of Transportation: It is the rating assigned by the respondent to public transportation, on a Likert-type scale with 5 options: very good, good, regular, bad, and very bad.

Air Quality: From the website of the CDMX Atmospheric Monitoring System, <http://www.aire.cdmx.gob.mx>, we obtained a year-long series of concentrations of particles less than 10 micrometers (PM10) at every monitoring station; then, we calculated the series of 24-hour mobile averages. Finally, following the calculations methodology of the Atmospheric Monitoring System Air Quality Index, we rated the air quality as “good”, “regular”, “bad”, “very bad”, and “extremely bad”. The “air quality” index used was determined as the percentage of days on which air quality was “good” or “regular.”

Safety in the Neighborhood: It is the response to the question, do you believe that living in your neighborhood is very safe, safe, neither safe nor unsafe, unsafe, or very unsafe?

Absence of Crime Victims: We considered crime such as auto theft, home theft, public transportation theft, cloning of a bank card, fraud, extortion, verbal threats, kidnapping, rape, forced disappearance, and others.

**References**

CONEVAL (2014). Metodología para la Medición Multidimensional de la Pobreza

enMéxico.México: Consejo Nacional de Evaluación de la Política de Desarrollo Social.

**
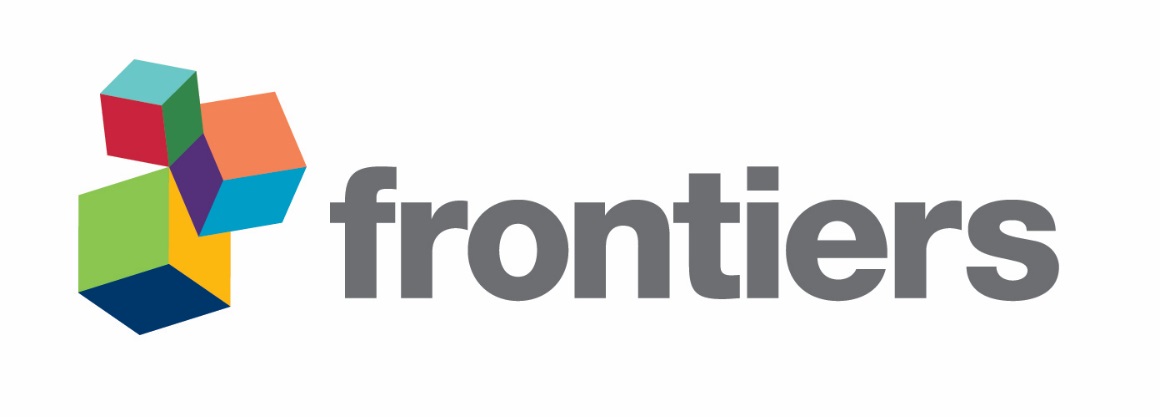
**

1. Mexico’s General Education Law calls basic compulsory education preschool, elementary school, and secondary school. [↑](#footnote-ref-1)
2. Social security is the right to medical services as a work benefit, as well as paid workers’ compensation in the case of an accident, illness, or maternity leave, and access to a contributory or non-contributory retirement or pension system (CONEVAL, 2014). [↑](#footnote-ref-2)
